# Argon Laser Phototherapy as an Adjunctive Treatment for Refractory Fungal Keratitis: A Retrospective Case Series

**DOI:** 10.7759/cureus.98465

**Published:** 2025-12-04

**Authors:** Astrid Pabon, Norka Sánchez, Salomon Merikansky, Guillermo Amescua, C Rocha-de-Lossada

**Affiliations:** 1 Ophthalmology, Centro de Investigaciones y Tratamiento Ocular, Buenos Aires, ARG; 2 Ophthalmology, OftalmoSalud, Lima, PER; 3 Ophthalmology, Bascom Palmer Eye Institute, University of Miami Miller School of Medicine, Miami, USA; 4 Ophthalmology, University of Miami Miller School of Medicine, Miami, USA; 5 Ophthalmology, Vithas Almería Hospital, Almeria, ESP

**Keywords:** amniotic membrane, argon laser, fungal keratitis, intrastromal voriconazole, pack-cxl (comparison), phototherapy, therapeutic keratoplasty

## Abstract

Background

This study aimed to evaluate the safety and clinical efficacy of argon laser phototherapy (ALP) as an adjunctive treatment for refractory fungal keratitis (FK) in a retrospective case series.

Methodology

This retrospective case series included 10 eyes from 10 patients with culture-proven FK that failed to improve after ≥2 weeks of topical antifungals. All eyes underwent adjunctive ALP (532 nm, 200 µm, 0.1 seconds, 900-1200 mW) applied to the fluorescein-stained ulcer bed until mild stromal blanching/microcavitation was observed. Standard topical antifungals were continued thereafter. The primary outcome was the time to complete epithelial healing with resolution of stromal infiltration. Secondary outcomes were the change in best-corrected visual acuity (BCVA) and adverse events. Minimum follow-up was three months.

Results

The mean age of the patients was 48.2 ± 11.6 years. Causative organisms included *Fusarium* (n = 5), *Aspergillus* (n = 3), and *Candida* (n = 2). Complete epithelial closure and resolution of infiltration were achieved in nine (90%) eyes within two to four weeks (mean ± SD = 2.6 ± 0.8 weeks). One *Fusarium* ulcer required amniotic membrane grafting for final closure. The mean BCVA improved from 0.2 to 0.4 (decimal) at final follow-up (p = 0.02). No cases of corneal melting, perforation, endothelial damage, or recurrence were observed during a mean follow-up of 4.2 ± 1.5 months.

Conclusions

Adjunctive ALP is a safe, effective, and accessible technique for refractory FK. Its combination of localized fungicidal effect and enhanced stromal drug penetration may accelerate healing, improve visual outcomes, and reduce the need for surgical intervention.

## Introduction

Fungal keratitis (FK) is a severe and potentially sight-threatening corneal infection that remains a major public health problem, especially in tropical and developing regions [1,2]. The disease is often associated with trauma from organic material, contact lens wear, or ocular surface disease, accounting for up to 60% of microbial keratitis cases in some endemic regions [3,4]. Despite advances in antifungal pharmacotherapy, the prognosis of FK remains guarded due to delayed diagnosis, poor drug penetration through intact epithelium and dense stroma, and the fungistatic rather than fungicidal action of most available agents. Conventional treatment relies mainly on topical polyenes, such as natamycin or amphotericin B, and on azoles, such as voriconazole or itraconazole. Yet, clinical failure rates remain high, with many cases progressing to corneal perforation or requiring keratoplasty [5,6].

Several adjunctive and experimental modalities have been proposed to enhance antifungal efficacy and shorten healing time, including intrastromal injection of voriconazole, corneal cross-linking (CXL), particularly photoactivated chromophore for keratitis-corneal cross-linking (PACK-CXL), and amniotic membrane grafting (AMG) [7-10]. However, despite these therapeutic advances, treatment outcomes remain inconsistent, and many cases still progress to corneal thinning or perforation. In this context, argon laser phototherapy (ALP) has emerged as a potential adjunctive approach capable of delivering localized fungicidal activity and improving stromal drug penetration, offering a simple and accessible alternative for nonhealing fungal ulcers [11,12].

The first clinical use of argon laser for refractory FK was reported by Pellegrino and Carrasco [12], demonstrating rapid resolution of *Fusarium* keratitis without adverse effects. Later, Khater et al. [13] conducted a prospective study showing that adjunctive argon laser therapy led to faster healing and improved visual outcomes compared with conventional antifungal therapy alone. A subsequent comparative trial by the same group reported that ALP achieved a 90% complete-healing rate within two to four weeks, significantly outperforming intrastromal voriconazole injection [13].

Based on previous clinical observations, we hypothesized that ALP could accelerate healing and improve outcomes in cases of FK unresponsive to conventional antifungal therapy. Therefore, the objective of this study was to evaluate the safety and clinical efficacy of ALP as an adjunctive treatment for refractory FK, including its therapeutic parameters, healing patterns, and potential advantages over standard medical management.

## Materials and methods

Study design and setting

This was a retrospective case series conducted at the Centro de Investigaciones y Tratamiento Ocular (CITO), Buenos Aires, Argentina, between January 2023 and December 2024. The study adhered to the principles of the Declaration of Helsinki. Given the retrospective design and the use of de-identified data, the committee waived the requirement for informed consent. Written consent for publication of clinical images was obtained from the corresponding patients.

Patient selection

Patients with culture- or smear-proven FK who remained unresponsive to at least two weeks of conventional antifungal therapy were enrolled. Inclusion criteria were (1) presence of an active corneal ulcer with stromal infiltration and epithelial defect; (2) positive identification of fungal elements on direct microscopy or culture; and (3) absence of clinical improvement despite maximal tolerated antifungal treatment.

Unresponsive infection was defined as the absence of clinical improvement after ≥2 weeks of maximal tolerated topical antifungal therapy, characterized by one or more of the following: (1) no reduction or enlargement of the epithelial defect; (2) persistence or increase in stromal infiltrate density or size; (3) new or worsening hypopyon; or (4) worsening pain or conjunctival inflammation.

Exclusion criteria were (1) corneal perforation or impending perforation; (2) presence of endophthalmitis; (3) previous corneal surgery within six months; or (4) uncontrolled systemic disease that could affect healing.

Baseline assessment

All patients underwent comprehensive ophthalmic evaluation, including slit-lamp biomicroscopy, fluorescein staining, anterior-segment optical coherence tomography, and best-corrected visual acuity (BCVA) measurement using the Snellen chart. Ulcer size, depth of stromal involvement, and presence of hypopyon were documented. The causative fungal species and antifungal sensitivity were determined by microbiological culture and staining techniques.

Conventional antifungal therapy

Before ALP, all patients received standard medical management consisting of topical natamycin 5%, amphotericin B 0.15%, or voriconazole 1%, selected according to culture and sensitivity results. Lubricants and cycloplegic drops were used for supportive care. Topical corticosteroids and systemic antifungals were withheld during the acute infection phase. ALP was considered only after failure to improve with this regimen for at least two weeks.

Argon laser phototherapy protocol

All procedures were performed under topical anesthesia with oxybuprocaine hydrochloride 0.4%. After instillation of fluorescein sodium 0.25%, the stained ulcer area was irradiated using an argon laser (532 nm; Carl Zeiss LSL 532s AG, Jena, Germany). The laser parameters were as follows: spot size, 200 µm; exposure time, 0.1 seconds; and power, 900-1,200 mW, adjusted according to tissue response. Laser shots were applied in a contiguous pattern covering the entire epithelial defect and infiltrated zone until mild stromal blanching and microcavitation bubbles were observed.

The number of pulses per session ranged between 20 and 150, depending on ulcer size. The procedure was performed once per patient. Post-laser care included topical antifungal continuation and preservative-free lubricants.

Follow-up and outcome measures

Patients were followed daily for the first week and then every three to five days until epithelial healing was complete. The primary outcome was time to complete epithelial closure and resolve stromal infiltration. Secondary outcomes included change in BCVA (lines gained or lost) and the need for AMG or keratoplasty. Recurrence or complications were also recorded. Follow-up continued for at least three months after clinical resolution.

Visual acuity reporting

BCVA was recorded in decimal notation. For reference, we included the corresponding decimal-to-logMAR conversions for the values used in this study (0.1 = 1.0 logMAR, 0.2 = 0.7 logMAR, 0.3 = 0.52 logMAR, 0.4 = 0.40 logMAR, 0.5 = 0.30 logMAR).

Statistical analysis

Descriptive statistics are expressed as mean ± standard deviation for continuous variables and percentages for categorical variables. Comparisons between pre- and post-treatment data were performed using the Wilcoxon signed-rank test or chi-square test, as appropriate. A p-value <0.05 was considered statistically significant. Statistical analyses were conducted using SPSS software version 25.0 (IBM Corp., Armonk, NY, USA).

## Results

A total of 10 eyes from 10 patients (six males and four females; mean age = ± SD: 48.2 ± 11.6 years) with culture-proven FK refractory to conventional antifungal therapy were included. The causative organisms were *Fusarium* spp. (n = 5), *Aspergillus* spp. (n = 3), and *Candida* spp. (n = 2). All patients had previously received topical natamycin 5% and/or amphotericin B 0.15% for at least two weeks with no significant improvement. Baseline demographic, microbiological, and clinical characteristics of all included eyes are summarized in Table 1.

**Table 1:** Clinical characteristics and outcomes of patients with refractory fungal keratitis treated with adjunctive argon laser phototherapy. BCVA = best-corrected visual acuity; CF = counting fingers; HM = hand motion

Case	Age/Sex	Etiologic agent	Ulcer location/size (mm)	Depth of infiltration	Pre-laser BCVA	Time from diagnosis to laser (days)	Laser parameters (532 nm, 200 µm, 0.1 s)/Number of spots	Post-laser epithelial healing (weeks)	Final BCVA	Complications/Recurrence
1	45/Male	*Fusarium* spp.	Central, 4.0 × 4.5	Anterior–mid stroma	CF	18	1,000 mW/70	3	0.4	None
2	52/Female	*Aspergillus* spp.	Paracentral, 3.5 × 3.5	Mid stroma	CF	20	1,100 mW/60	2	0.5	None
3	39/Male	*Candida* spp.	Inferior, 3.0 × 3.0	Anterior stroma	0.2	15	900 mW/45	2	0.6	None
4	54/Male	*Fusarium* spp.	Central, 5.0 × 4.5	Mid stroma	HM	21	1,100 mW/25	4	0.3	None
5	60/Female	*Aspergillus* spp.	Paracentral, 4.0 × 3.0	Anterior stroma	CF	16	1,000 mW/97	3	0.4	None
6	47/Male	*Fusarium* spp.	Central, 4.5 × 4.0	Mid stroma	CF	17	1,200 mW/90	3	0.4	None
7	44/Female	*Candida* spp.	Paracentral, 3.5 × 3.0	Anterior stroma	0.1	14	900 mW/50	2	0.5	None
8	59/Male	*Aspergillus* spp.	Central, 4.2 × 4.0	Anterior–mid stroma	HM	19	1,000 mW/80	2	0.4	None
9	50/Male	*Fusarium* spp.	Central, 4.0 × 3.8	Mid stroma	CF	18	1,100 mW/75	3	0.5	None
10	63/Female	*Fusarium* spp.	Inferior, 3.0 × 2.8	Anterior stroma	CF	20	900 mW/40	4	0.3	None

Baseline characteristics

At presentation, the mean ulcer diameter was 4.3 ± 1.1 mm, with stromal infiltration depth involving the anterior-to-mid stroma in all cases. A hypopyon was observed in six (60%) eyes. The baseline BCVA ranged from counting fingers to 0.2 (decimal system).

Response to argon laser phototherapy

**Figure 1:**
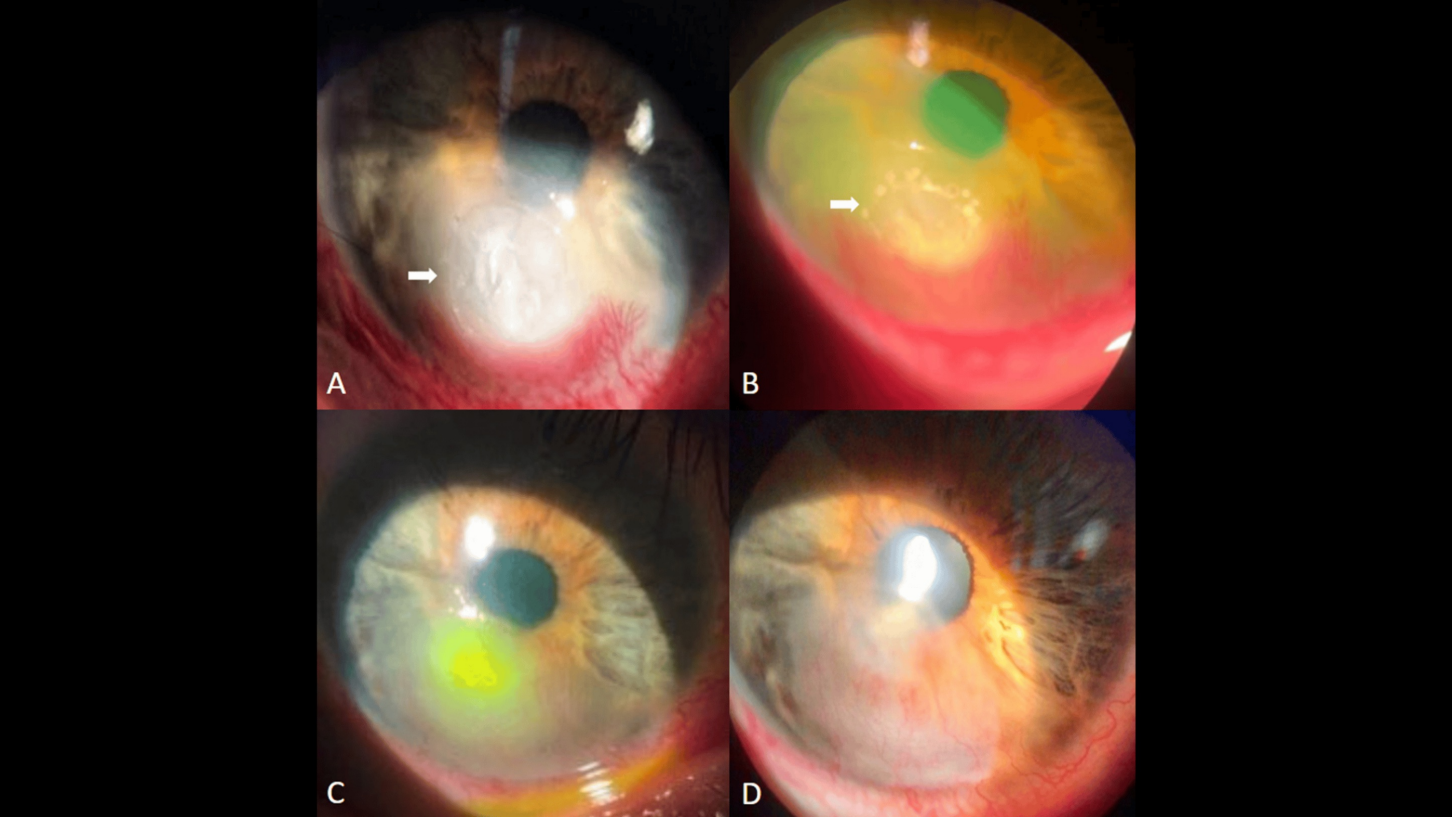
Representative case of Fusarium keratitis treated with adjunctive ALP. (A) Baseline slit-lamp image showing dense central stromal infiltrate and epithelial defect (white arrows) with conjunctival hyperemia. (B) Immediate post-laser fluorescein view after ALP (532 nm, 200 µm, 0.1 s, ~1,000 mW) demonstrating mild stromal blanching and microcavitation at the ulcer bed (white arrows). (C) Early re-epithelialization at one week with decreased stromal haze. (D) Complete epithelial healing by week four with residual stromal opacity. Arrows indicate the area of clinical interest in all subimages. ALP = argon laser phototherapy

**Figure 2:**
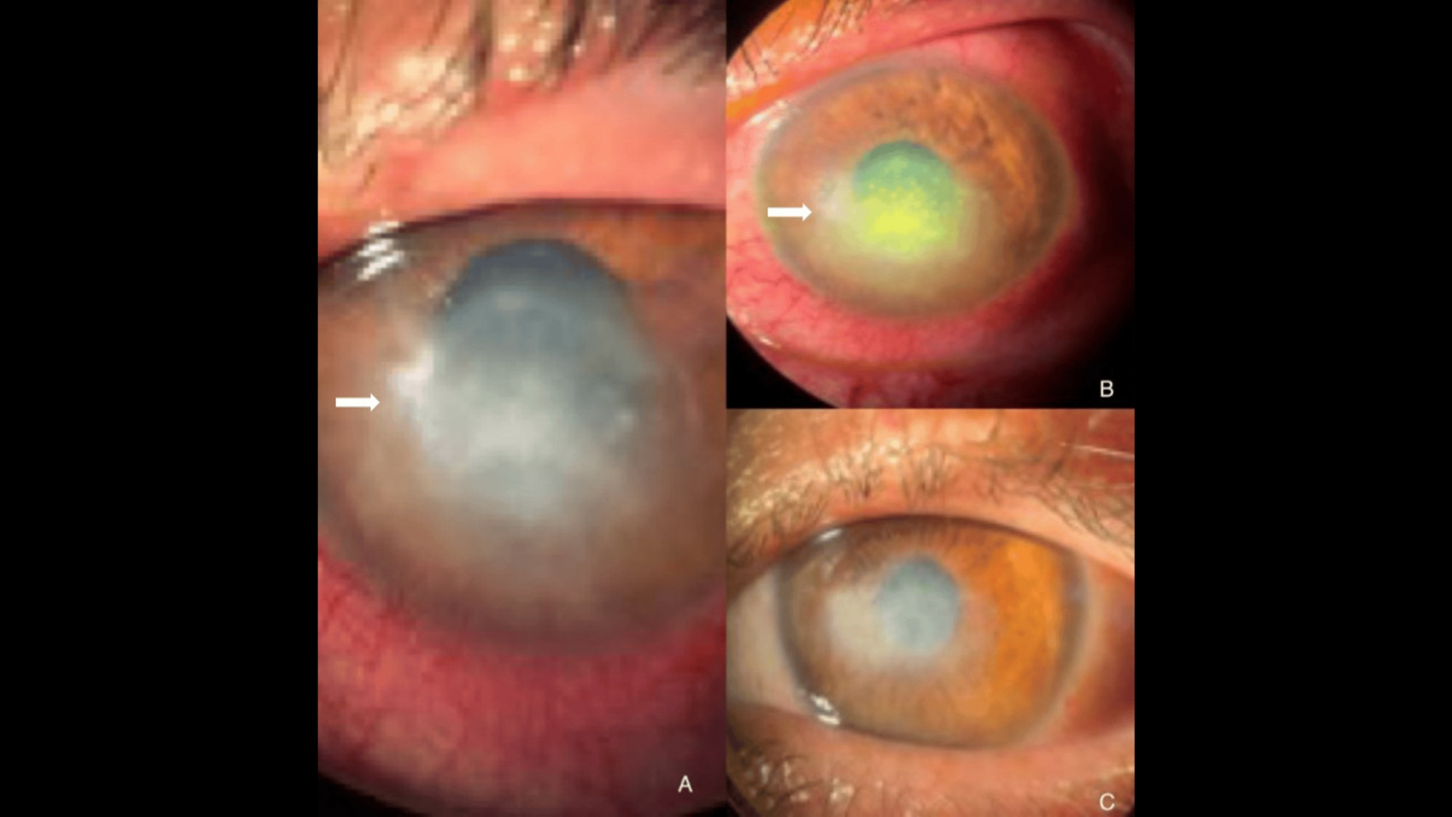
Healing patterns across different etiologies after ALP. (A) Baseline *Aspergillus* keratitis with extensive stromal opacity and edema (white arrows). (B) Immediate post-laser fluorescein view highlighting the treated zone and mild stromal blanching (white arrows). (C) Four-week follow-up from another case showing complete epithelialization and reduced stromal opacity. Arrows indicate the area of clinical interest in all subimages. ALP = argon laser phototherapy

Following argon laser application, all patients demonstrated a rapid decrease in stromal infiltration and epithelial defect size within the first week. Complete epithelial closure and resolution of infiltration were achieved in 9 of 10 (90%) eyes within two to four weeks (mean ± SD = 2.6 ± 0.8 weeks). One patient with a deep *Fusarium* ulcer required AMG to achieve complete healing by week six. Representative slit-lamp photographs show progressive re-epithelialization and reduction in stromal opacity after laser treatment (Figures 1A-1D; Figures 2A-2C).

Visual outcomes

Post-treatment BCVA improved by at least two or more lines in four (40%) eyes, by one line in three (30%) eyes, and remained stable in three (30%) eyes. No patient experienced deterioration of visual acuity. The mean final BCVA was 0.4 ± 0.2, significantly better than baseline (p = 0.02, Wilcoxon signed-rank test).

Complications and recurrence

**Figure 3:**
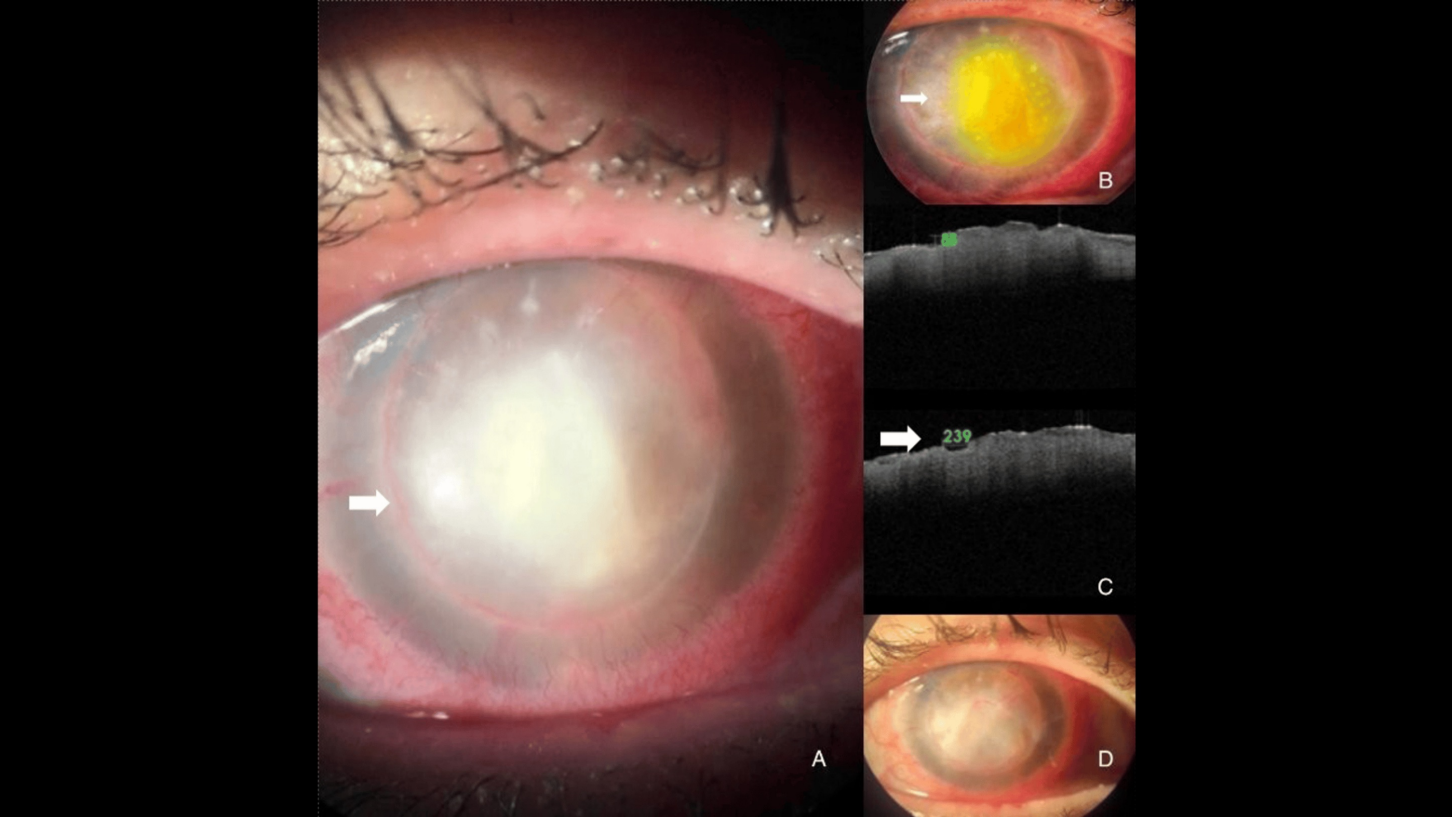
Clinical and AS-OCT correlation following ALP. (A) Baseline slit-lamp image with a 6 × 7-mm central stromal infiltrate and epithelial defect (white arrows). (B) Immediate post-laser appearance of the infiltrate (white arrows). (C) Upper: AS-OCT cross-section showing anterior-to-mid stromal hyperreflectivity and increased stromal thickness before treatment; Lower: AS-OCT from the same eye immediately after ALP, demonstrating reduced anterior stromal hyperreflectivity and early tissue compaction (white arrow). (D) Two-week clinical resolution with restored epithelial continuity and decreased stromal reflectivity. Arrows indicate the area of interest in all subimages. AS-OCT = anterior segment optical coherence tomography; ALP = argon laser phototherapy

No adverse events related to the laser procedure, such as corneal melting, perforation, endothelial damage, or secondary infection, were observed. Tomographic correlation confirmed reduced anterior stromal hyperreflectivity and thickness after treatment (Figure 3). No recurrence was detected during the mean follow-up period of 4.2 ± 1.5 months.

## Discussion

When evaluating adjunctive treatments for refractory FK, ALP should be considered part of a broader therapeutic strategy to enhance antifungal efficacy and promote corneal healing. Over the past decade, several interventions have been investigated with varying success. Despite these developments, the management of deep or recalcitrant fungal ulcers remains difficult, as drug diffusion through the corneal stroma is poor and fungal biofilms or enzymatic degradation further compromise medical response [14]. Consistent with previous reports showing 80-90% healing within two to four weeks using ALP, our findings further support the efficacy and safety of this adjunctive approach.

Among these options, intrastromal voriconazole injection has gained popularity as a means of delivering high drug concentrations directly to the site of infection. Sharma et al. and Prakash et al. reported encouraging outcomes in deep fungal ulcers, achieving resolution in approximately 80% of cases after one or more injections [15,16]. Nevertheless, subsequent studies, including the randomized comparative trial by Khater et al., demonstrated that intrastromal injections may require repeated administration, carry the risk of mechanical trauma to friable corneal tissue, and are less effective against *Fusarium* infections due to innate resistance [13]. In contrast, the argon laser group in the same study achieved faster and more consistent healing, with complete resolution in 90% of eyes within two to four weeks, highlighting that ALP appears promising relative to other adjunctive options in terms of efficacy and reproducibility.

PACK-CXL has also been evaluated as an adjuvant in infectious keratitis, leveraging the generation of reactive oxygen species to induce microbial killing and collagen stiffening. Galperin et al. reported initial success using UVA-riboflavin cross-linking in *Fusarium* keratitis, but subsequent studies revealed inconsistent efficacy, limited penetration depth of UVA light, and potential toxicity to the endothelium and lens [17]. Compared with CXL, ALP provides focal and controlled energy delivery, targeting the infected stroma without exposing the entire cornea to ultraviolet irradiation. Moreover, the thermal and microcavitation effects induced by the 532-nm argon beam produce immediate stromal sterilization and microstructural remodeling [12,13,18], mechanisms distinct from and arguably more predictable than those of photooxidative cross-linking.

AMG remains another important adjunct for ocular surface reconstruction and control of inflammation in advanced FK. Yildiz et al. and Sandinha et al. demonstrated that AMG can support epithelial healing and reduce pain in nonhealing ulcers [19,20]; however, it does not directly address the infectious burden. Khater et al. later demonstrated that combining AMG with ALP yielded superior structural outcomes and faster epithelialization compared with AMG alone, likely due to a synergistic effect of initial fungal load reduction followed by stromal coverage and cytokine modulation [13]. In our experience, only one patient required AMG after ALP, underscoring that ALP alone may obviate the need for more invasive procedures in many cases.

Other options, such as Rose Bengal photodynamic therapy, cyanoacrylate gluing, or therapeutic keratoplasty [21-25], may offer structural benefit in select cases but are generally invasive and reserved for advanced disease. In contrast, ALP is minimally invasive, can be performed at the slit lamp under topical anesthesia, and utilizes equipment readily available in most ophthalmic units. The ability to directly observe tissue blanching and microcavitation during treatment ensures controlled energy delivery. The absence of serious complications across studies, including the present series, further reinforces its safety profile.

While these advantages position ALP favorably among emerging adjuvant therapies, the present findings should be interpreted in light of certain limitations. The relatively small sample size and single-center design limit the generalizability of our findings and should be taken into account when interpreting the results. The number of eyes included was restricted to patients who fulfilled stringent inclusion criteria, namely culture-proven FK refractory to at least two weeks of antifungal therapy, reflecting the low incidence yet high clinical complexity of this condition. In addition, the absence of a control group treated exclusively with medical therapy prevents direct statistical comparison between modalities. The possibility of regression to the mean, inherent to a disease with fluctuating inflammatory activity, must also be considered. Furthermore, due to the retrospective design, outcome assessment was not masked, introducing potential observer bias. The relatively short follow-up period may underestimate late recurrence. Nevertheless, our outcomes are consistent with those reported in larger prospective trials by Khater et al. [13]. Finally, the lack of histopathologic correlation or in vivo confocal microscopy limits insight into the precise cellular-level structural changes induced by laser energy. Future experimental models integrating advanced imaging may further elucidate the biomechanical and pharmacokinetic mechanisms underpinning the observed clinical response.

Despite these limitations, the findings offer several important clinical implications. Argon laser phototherapy appears particularly beneficial in cases characterized by superficial-to-mid-stromal infiltration, where conventional antifungal agents exhibit limited diffusion and surgical intervention may otherwise be required. The rapid epithelial closure and stromal sterilization achieved in our patients suggest that this technique can reduce the duration of medical therapy, minimize the risk of perforation, and possibly defer or prevent keratoplasty in selected cases. Moreover, the absence of intraoperative or postoperative complications, such as corneal melting or endothelial toxicity, reinforces the procedure’s safety and reproducibility when performed under controlled parameters. Importantly, this technique can be implemented in most ophthalmic centers equipped with a standard retinal argon laser, expanding its potential applicability in both tertiary and regional hospitals, especially in low-resource settings where FK remains a leading cause of monocular blindness.

## Conclusions

From a therapeutic standpoint, ALP may fill a useful gap between purely pharmacologic and surgical management. Its dual mechanism, direct photothermal fungicidal activity combined with enhanced stromal penetration of antifungal agents, offers a logical, targeted approach to the pathophysiology of refractory FK. By addressing both the microbial and microstructural components of the disease, ALP aligns with current trends toward minimally invasive, pathogen-specific interventions aimed at preserving corneal integrity and optimizing recovery. Future multicenter studies with randomized controlled designs and standardized laser parameters are needed to validate these findings and establish definitive treatment guidelines. In summary, our results suggest that ALP may serve as a safe, effective, and accessible adjunctive therapy for refractory FK. By potentially accelerating epithelial healing, improving visual outcomes, and reducing the need for surgical intervention, ALP may become an important component of the therapeutic armamentarium against severe fungal keratitis, particularly in settings with limited resources.
